# LT-IIc, A Bacterial Type II Heat-Labile Enterotoxin, Induces Specific Lethality in Triple Negative Breast Cancer Cells by Modulation of Autophagy and Induction of Apoptosis and Necroptosis

**DOI:** 10.3390/ijms20010085

**Published:** 2018-12-26

**Authors:** Patricia Masso-Welch, Sofia Girald Berlingeri, Natalie D. King-Lyons, Lorrie Mandell, John Hu, Christopher J. Greene, Matthew Federowicz, Peter Cao, Terry D. Connell, Yasser Heakal

**Affiliations:** 1 Department of Biotechnical and Clinical Laboratory Sciences, Jacobs School of Medicine and Biomedical Sciences, University at Buffalo, 3435 Main Street, Buffalo, NY 14214, USA; pmwelch@buffalo.edu (P.M.-W.); sofia.girald@upr.edu (S.G.B.); 2 Department of Pharmaceutical, Social and Administrative Sciences, School of Pharmacy, D’Youville College, 320 Porter Avenue, Buffalo, NY 14201, USA; federm07@dyc.edu (M.F.); caop13@dyc.edu (P.C.); 3 Department of Microbiology and Immunology and the Witebsky Center for Microbial Pathogenesis and Immunology, Jacobs School of Medicine and Biomedical Sciences, The University at Buffalo, 955 Main Street, Buffalo, NY 14203, USA; ndking@buffalo.edu (N.D.K.-L.); lmc29@buffalo.edu (L.M.); conghu@buffalo.edu (J.H.); christopher.greene@roswellpark.org (C.J.G.); connell@buffalo.edu (T.D.C.)

**Keywords:** breast cancer, bacterial enterotoxins, heat-labile enterotoxins, autophagy, apoptosis, necroptosis, triple-negative breast cancer, LT-IIc

## Abstract

Triple negative breast cancer (TNBC) remains a serious health problem with poor prognosis and limited therapeutic options. To discover novel approaches to treat TNBC, we screened cholera toxin (CT) and the members of the bacterial type II heat-labile enterotoxin family (LT-IIa, LT-IIb, and LT-IIc) for cytotoxicity in TNBC cells. Only LT-IIc significantly reduced viability of the TNBC cell lines BT549 and MDA-MB-231 (IC_50_ = 82.32 nM). LT-IIc had no significant cytotoxic effect on MCF10A (IC_50_ = 2600 nM), a non-tumorigenic breast epithelial cell line, and minimal effects on MCF7 and T47D, ER^+^ cells, or SKBR-3 cells, HER2^+^ cells. LT-IIc stimulated autophagy through inhibition of the mTOR pathway, while simultaneously inhibiting autophagic progression, as seen by accumulation of LC3B-II and p62. Morphologically, LT-IIc induced the formation of enlarged LAMP2+ autolysosomes, which was blocked by co-treatment with bafilomycin A1. LT-IIc induced apoptosis as demonstrated by the increase in caspase 3/7 activity and Annexin V staining. Co-treatment with necrostatin-1, however, demonstrated that the lethal response of LT-IIc is elicited, in part, by concomitant induction of necroptosis. Knockdown of ATG-5 failed to rescue LT-IIc-induced cytotoxicity, suggesting LT-IIc can exert its cytotoxic effects downstream or independently of autophagophore initiation. Collectively, these experiments demonstrate that LT-IIc acts bifunctionally, inducing autophagy, while simultaneously blocking autolysosomal progression in TNBC cells, inducing a specific cytotoxicity in this breast cancer subtype.

## 1. Introduction

Triple-negative breast cancer (TNBC) is characterized by the lack of detectible expression of estrogen receptor (ER), progesterone receptor (PR), and human epidermal growth factor receptor 2 (HER2/Neu/ErbB2) [1]. Although TNBC accounts for only 15–20% of breast cancer, patients diagnosed with TNBC are at higher risk of disease recurrence and progression [1]. Despite the advances in the treatment of breast cancer, TNBC remains a particularly lethal disease, with only limited options for effective treatment [1]. TNBC patients with metastatic disease typically receive chemotherapy and radiation, a clinical strategy that has limited efficacy due to metastasis and resistance that could be caused, in-part, by resistant cancer stem cells [2]. Historically, a number of unconventional treatments for various cancers have been attempted. These treatments have included bacteria and bacterial toxins [3]. More recently, Havas and Donnelly experimented with the use of mixed bacterial toxins to treat mouse models of cancer [4]. The lack of reproducible patient response and mechanistic understanding of these approaches, however, has been a major hurdle for the advancement of bacterial toxins into the clinic as therapeutic agents for cancer treatment.

Autophagy has emerged as a promising target for cancer intervention [5]. The conflicting roles of autophagy in protection against, versus promotion of, cancer, however, remain to be defined to optimally advance this field to the clinic [6]. As TNBC cells have been shown to be dependent on activated autophagy for survival even under nutrient-rich conditions, inhibition of autophagy and/or its downstream targets, such as lysosomal degradation, could be a plausible strategy for development of novel therapeutics to treat this subtype of breast cancer [7,8]. Additionally, breast cancer stem cells are also dependent on autophagy for their maintenance and survival [9,10,11]. Other studies, however, demonstrated that stimulating autophagy could lead to cell death, possibly by induction of apoptosis or independently by inducing autophagic cell death [12]. These findings suggest that inducing autophagy, while simultaneously inhibiting its progression, could be an effective approach to lethally target TNBC and/or cancer stem cells.

Recently, we observed that LT-IIc, a member of the type II subfamily of heat-labile enterotoxins (HLT) of *Escherichia coli* [13,14], induced cell death in murine TNBC cell lines. Death was concomitant with the rapid accumulation of extensive intracellular large vacuoles. Herein, we examined the effects of LT-IIc and other bacterial enterotoxins on a panel of human breast cancer cell lines. LT-IIc induced the accumulation of enlarged LAMP-2 positive autolysosomes [15,16] and upregulation of LC3B-II and p62 (sequestosome) [17], thereby indicating an inhibition of autophagic progression in those cells. This inhibition occurred concomitant with mTOR-dependent stimulation of autophagy. Interestingly, LT-IIc treatment caused a robust induction of caspase 3/7 activity, thus indicating induction of apoptosis. Co-treatment with the pan-caspase inhibitor, Z-VAD-FMK [18] partially rescued MDA-MB-231 cell survival. Treatment of cells with necrostatin-1, a necroptosis inhibitor [19], enhanced the rescue of cell viability; co-treatment of the cells with Z-VAD-FMK and necrostatin-1 essentially elicited full rescue of cell viability. These data strongly demonstrated that LT-IIc mediated cell death by a combined induction of apoptosis and necroptosis. Knockdown of ATG5 by siRNA did not inhibit LT-IIc-mediated cytotoxicity, supporting the concept that LT-IIc can induce cytotoxicity through its effects on later stages, e.g., autolysosomal processing. Further studies of the unique cytotoxic effects of LT-IIc on TNBC cells will further validate LT-IIc as a novel therapeutic agent and will reveal novel “druggable” targets for treating this particularly lethal form of breast cancer.

## 2. Results

### 2.1. LT-IIc Selectively and Irreversibly Induces Cell Death of TNBC Cells 

**Figure 1:**
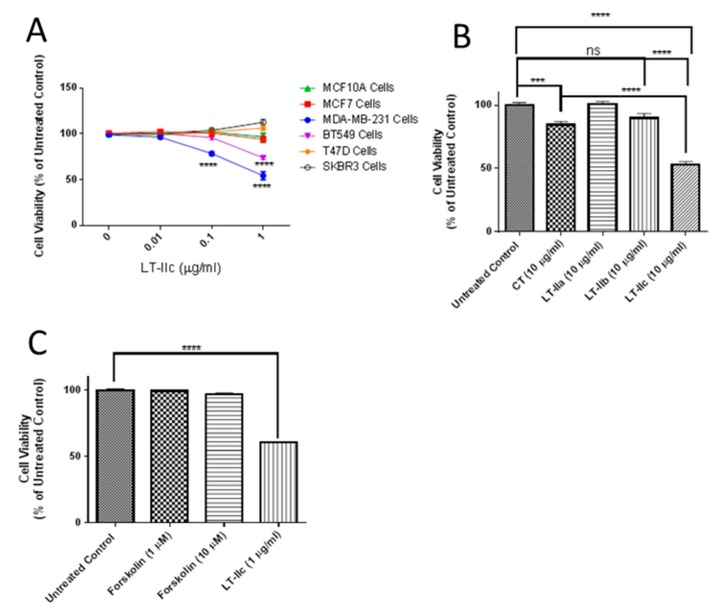
Effects of LT-IIc on breast cancer cell viability and morphology. (**A**) LT-IIc was specifically toxic to BT549 and MDA-MB-231 TNBC cell lines, but not MCF10A, MCF7, or SK-BR3 cells. All cell lines were treated with 0, 0.01, 0.1, 1, or 10 µg/mL LT-IIc for 48 h, followed by MTT assay. Data points represents the means ± SEM of three independent experiments. (**B**) LT-IIc, CT, LT-IIa, and LT-IIb (10 µg/mL) were tested for durable cytotoxic effects by pulsing MDA-MB-231 breast cancer cells for 24 h, followed by a wash-out period of an additional 24 h. Cytotoxicity, assessed using MTT, showed the greatest effect in cells treated with LT-IIc. Bars represent mean ± SEM from two independent experiments with eight replicates each. (**C**) The cytotoxic effects of LT-IIc enterotoxin were not mimicked by forskolin, an activator of adenylate cyclase. MDA-MB-231 cells were treated with LT-IIc (1 µg/mL) or forskolin (1 or 10 µM) for 48 h, followed by assessment of cell viability using MTT assay. The data represent the mean ± SEM of three replicates. Key: *** *p* < 0.001; **** *p* < 0.0001. ns (non-significant).

Previously, we observed that LT-IIc induced cell death in the murine 4T1 triple negative breast cancer cell line, and the less-characterized TM12T transformed mouse breast mesenchymal cell line, but not the parental pre-neoplastic epithelioid TM12 cells (unpublished data). To evaluate the cytotoxic specificity of LT-IIc towards various types of human breast cancer cells, we compared its effects on TNBC human breast cancer cell lines MDA-MB-231 and BT549, the ER^+^ breast cancer cell lines MCF-7 and T47D, the HER2^+^ breast cancer cell SKBR3, and MCF10A, an immortalized, but not transformed, breast epithelial cell line. After 48 h of treatment, cell lines were assessed for cell viability using (3-(4,5-Dimethylthiazol-2-yl)-2,5-Diphenyltetrazolium Bromide) (MTT) assay. LT-IIc induced a significant decrease in viable cell numbers at both 0.1 µg/mL and 1 µg/mL only in MDA-MB-231 and BT549, but not in the non-TNBC cells lines T47D, SKBR3, MCF7, or MCF10A (Figure 1A). To determine if these TNBC-specific lethal effects were unique to LT-IIc, or could be mimicked by type I heat-labile enterotoxin or other type II heat-labile enterotoxins, MDA-MB-231 cells were pulsed with cholera toxin (CT), LT-IIa, LT-IIb, or LT-IIc for 24 h after which the enterotoxins were removed and the cells incubated for an additional 24 h in toxin-free culture medium. Of the four enterotoxins, LT-IIc exhibited the most substantial enduring cytotoxic effect, reducing cell viability by ~50% (Figure 1B). 

### 2.2. Requirement for Adenylate Cyclase Activity for Cytotoxic Effects in MDA-MB-231 Cells 

Both type I and type II heat-labile enterotoxins (HLT) intoxicate cells by ADP-ribosylating the GSα regulatory protein of adenylate cyclase, thereby dramatically increasing production of cAMP, which activates cAMP-dependent downstream cellular responses [20]. Since CT, LT-IIa, and LT-IIb did not exert the same cytotoxic effects as LT-IIc, the specific lethal effects of LT-IIc for TNBC were likely not due to its ADP-ribosylase activity. To further evaluate this model, cells were treated with forskolin, a direct pharmacological activator of adenylate cyclase [21]. Treatment of MDA-MB-231 cells with forskolin at a range of concentrations known to activate adenylate cyclase and increase intracellular cAMP did not mimic the cytotoxicity (Figure 1C) or cellular morphology (described below) induced by LT-IIc, further confirming that the effects were not mimicked by elevation of intracellular cAMP. 

### 2.3. LT-IIc Induces Accumulation of Autolysosomes Specifically in TNBC

**Figure 2:**
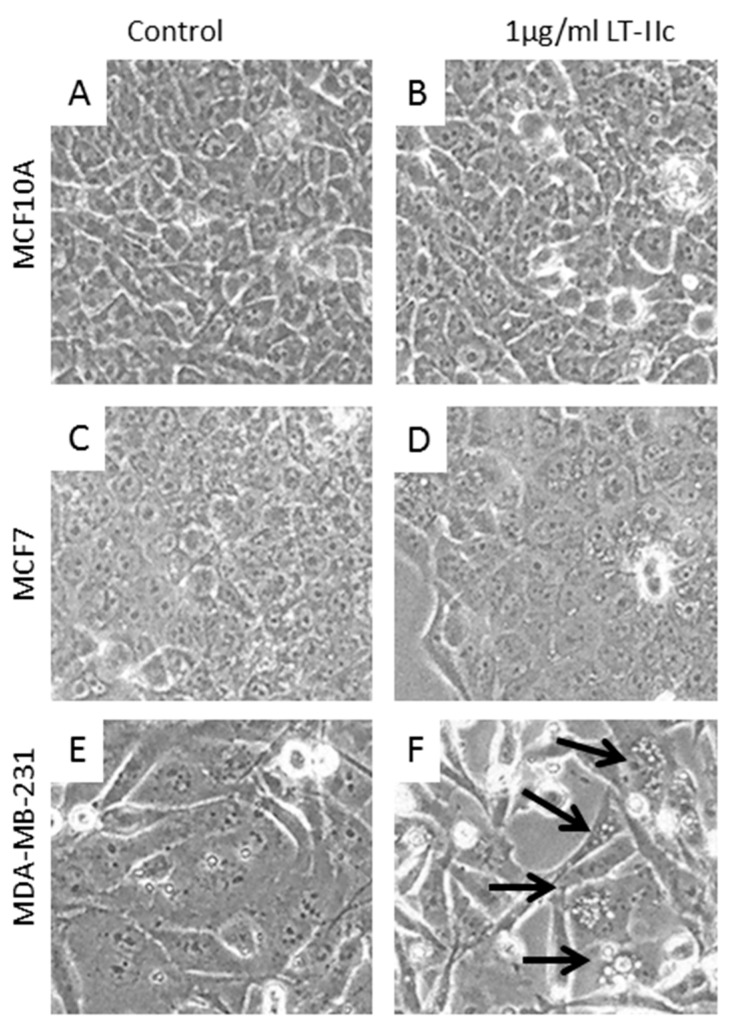
LT-IIc did not induce morphologic change in the immortalized human breast epithelial MCF10A (**A**,**B**), or ER positive MCF7 human breast cancer cell lines (**C**,**D**). LT-IIc induced extensive intracellular vacuolation (arrows) in MDA-MB-231 (**E**,**F**). All cells were treated with 1 µg/mL LT-IIc for 24 h. Cell images were obtained using a 40× objective under phase contrast illumination.

Treatment of MCF10A (Figure 2A,B), or MCF7 (Figure 2C,D) resulted in no apparent change in cellular morphology. In contrast, treatment of MDA-MB-231 cells with LT-IIc elicited a rapid and extensive formation of distended, phase bright vacuoles that were as large as 24 µm in diameter (Figure 2E,F). Many swollen floating cells were also observed that resembled necrotic cells. BT549 cells exhibited a similar morphologic response to LT-IIc (Supplementary Figure S1). Systematic analysis of the diameter of these vacuoles revealed that LT-IIc treatment induced vacuoles whose average size ranged from 8–13 µm in MDA-MB-231 cells, with a maximum diameter of 24 µm, and from 5–8 µm in BT549 cells, with a maximum diameter of 23 µm. Since autophagosome size normally ranges from 140–160 nm in diameter for cytoplasmic to vacuolar autophagosomes, or 300–900 nm in nitrogen starved cells [22], these data suggested an alternative origin, such as lipid droplets or swollen lysosomes. 

**Figure 3:**
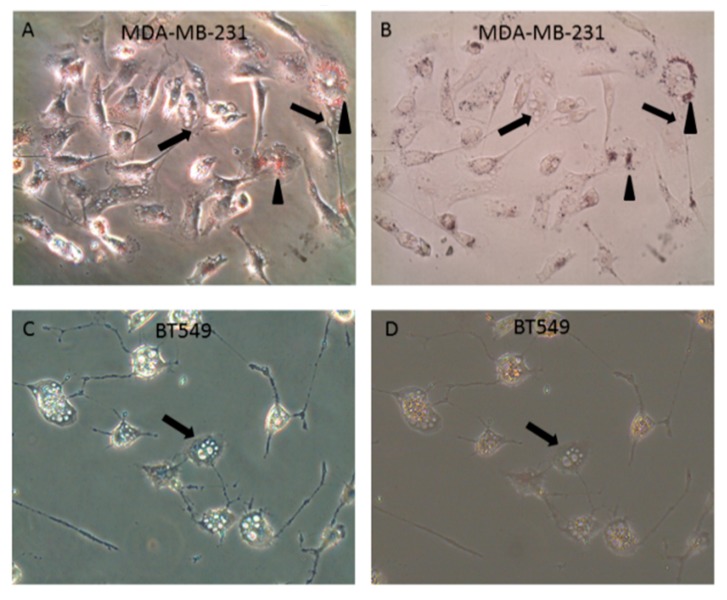
LT-IIc-induced vacuolation is not due to intracellular lipid accumulation. (**A**) Oil Red O staining revealed perinuclear pool of small lipid droplets (arrowheads) in all MDA-MB-231 cells treated with 1 µg/mL LT-IIc for 24 h, viewed under phase contrast (**A**) or bright field, after staining with Oil Red O (**B**). This small perinuclear lipid pool was independent of the large vacuoles (arrows). BT549 treated with 1 µg/mL LT-IIc lacked perinuclear lipid droplets seen in MDA-MB-231 cells. Large intracellular vacuoles were Oil Red O negative (arrows). (**C**,**D**) BT549 cells lacked perinuclear lipid droplets seen in MDA-MB-231 cells. Large intracellular vacuoles were oil red O negative. All images were obtained using 40× objective magnification.

To determine whether these vacuoles were the result of intracellular lipid accumulation, staining with Oil Red O was performed. Although MDA-MB-231 cells showed abundant small perinuclear vacuoles stained with Oil Red O, lack of staining of the distended vacuoles with Oil Red O demonstrated that these large bodies were not the result of intracellular lipid accumulation (Figure 3A,B). Similar to MDA-MB-231 cells, no association of Oil Red O staining with the distended intracellular vesicles (Figure 3C,D) were observed in BT549 cells. In both cell lines, any small Oil Red O-positive droplets (arrowheads) were distinctly separate from the large intracellular vacuoles (arrows). Therefore, these vacuoles were not due to mammary epithelial lactogenic differentiation, or other pathways involving accumulation of intracellular lipid. 

**Figure 4:**
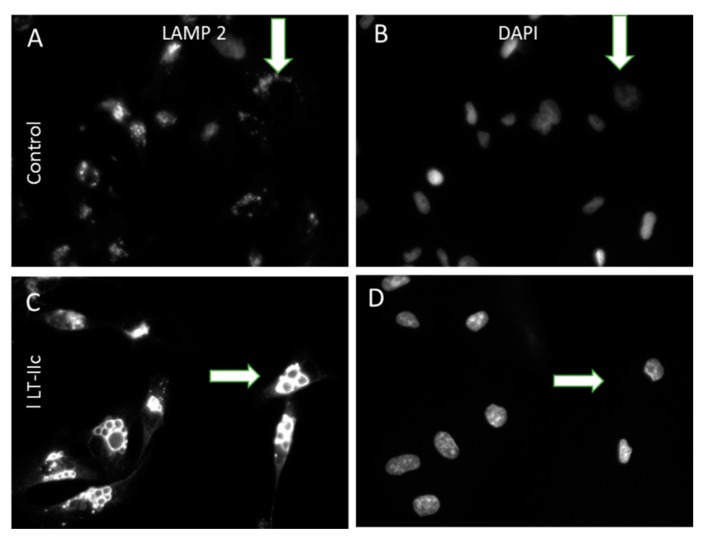
Enlarged intracellular vacuoles induced by LT-IIc are positive for LAMP-2. (**A**,**B**): Untreated MDA-MB-231 cells display punctate cytoplasmic staining for small lysosomes expressing LAMP-2 (**A**, arrow); DAPI staining for nuclei in same field (**B**). (**C**,**D**): LT-IIc-treated cells at 6 h possess multiple enlarged LAMP-2-stained bodies (**C**, arrow); DAPI staining of the same field (**D**). All images were taken under a 40× objective, using identical manual exposure times for control versus treated cells.

To determine if LT-IIc is targeting the autophagosomal-lysosomal pathway, we investigated whether these enlarged vacuoles could represent swollen lysosomes by staining for LAMP-2 (lysosome-associate membrane protein 2) [23]. Untreated MDA-MB-231 cells express many small cytoplasmic, punctate lysosomes stained with LAMP-2 (Figure 4A,B, arrows). In contrast, LT-IIc treatment induced very large swollen vacuoles that were intensely positive for LAMP-2 staining (Figure 4C,D, arrows). 

### 2.4. LT-IIc Alters Markers of Autophagic Flux in TNBC Cell Lines 

**Figure 5:**
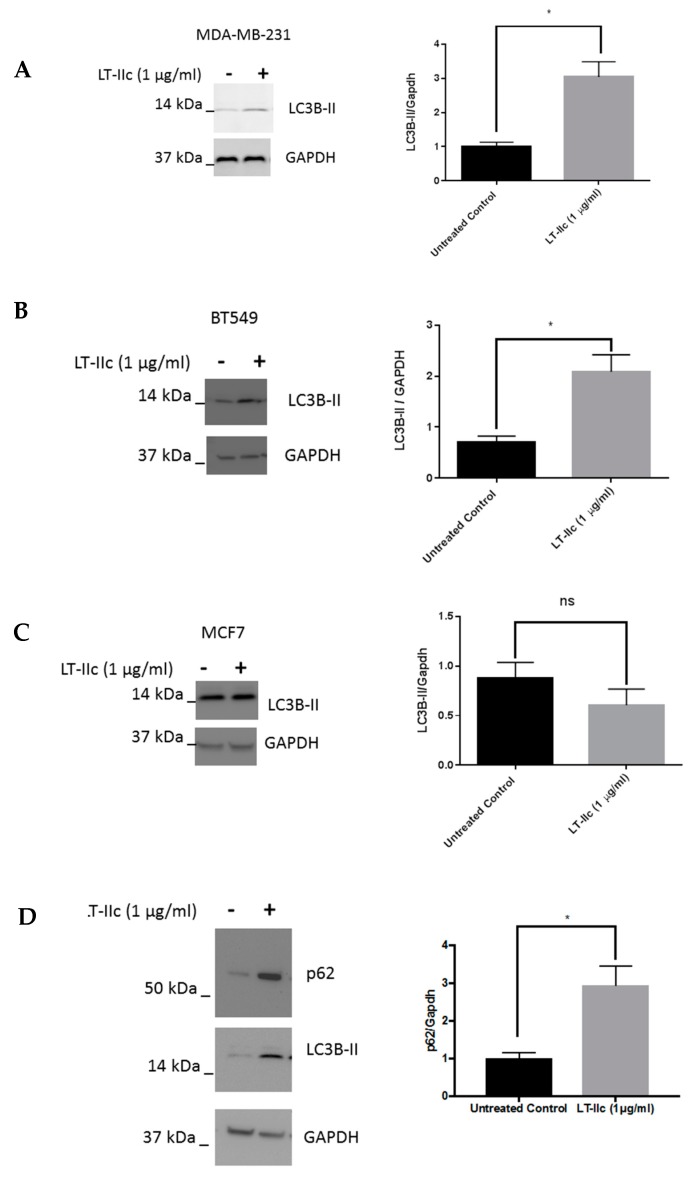
The effect of LT-IIc on autophagy in breast cancer cells. MDA-MB-231, BT549, and MCF7 cells were treated with 1 µg/mL LT-IIc for 24 h, prior to generating lysates for Western blotting. (**A**) Representative Western blot for LC3B-II with GAPDH as a loading control is shown (*N* = 3 independent experiments) for MDA-MB-231 (**A**), BT549 (**B**), and MCF7 cells (**C**). (**D**) Lysates from MDA-MB-231 cells treated ±1 µg/mL LT-IIc were analyzed for the expression of p62 protein levels by Western blotting, using GAPDH as a loading control. Representative blot of three independent experiments. Quantitation of blots was performed using Image J. Error bars represent SEM. Key: * *p* < 0.05.

**Figure 6:**
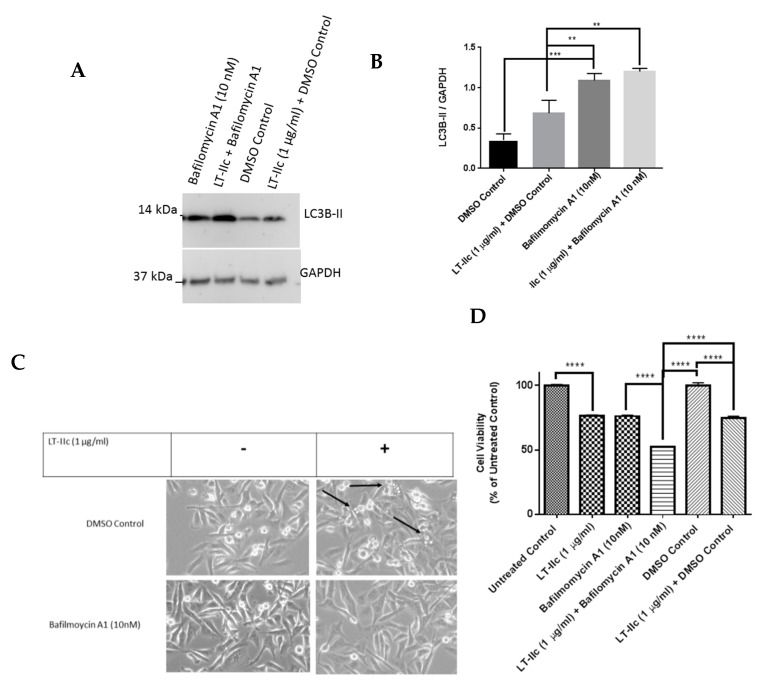
Effects of bafilomycin A1 on LT-IIc efficacy in MDA-MB-231 cells. (**A**) MDA-MB-231 cells were treated with LT-IIc (1 µg/mL) in the presence or absence of bafilomycin A1 (10 nM) for 48 h. (**B**) The level of expression of LC3B-II was analyzed using Western blotting and the blots were quantified using ImageJ software. (**C**) MDA-MB-231 cells were treated with LT-IIc (1 µg/mL) in the presence or absence of bafilomycin A1 (10 nM) for 24 h and cell morphology was evaluated using microscopic analysis (10× magnification). Arrows indicate vacuoles observed in LT-IIc treated cells not exposed to bafilomycin A1. (**D**) Bafilomycin A1 and LT-IIc showed similar effects on MDA-MB-231 cytotoxicity (measured by MTT assay). Co-treatment significantly enhanced cytotoxicity (compared to LT-IIc plus DMSO control). All panels represent at least three independent replicates from three independent experiments. Bars represent means ± standard error of the mean. ** *p* < 0.01; *** *p* < 0.0001; **** *p* < 0.00001.

The accumulation of swollen LAMP-2 positive lysosomes specifically in TNBC cells suggests that autophagic flux may be altered by treatment with LT-IIc (i.e., the levels of autophagy may be exceeding the degradative capacity of the lysosomes). To test this hypothesis, we evaluated the effects of LT-IIc on LC3B, a protein whose lipidated form (LC3B-II) is essential for autophagosome formation, and is broken down by autophagosomes proceeding through lysosomal fusion (to form the autolysosome) resulting in subsequent proteolytic degradation [24]. A single band for LC3B was detected in Figure 5 (and Figure 6). Based on the stronger reactivity of this antibody with the type II isoform of LC3B, it is possible that the non-lipidated LC3B-I protein was below the level of detection of this exposure. Treatment of MDA-MB-231 cells with LT-IIc increased the amount of LC3B-II by >150% (Figure 5A). Similar results were observed in BT-549 cells (Figure 5B). 

In contrast, no significant increase in the levels of LC3B-II were observed in MCF7 (ER+) human breast cancer cells after treatment with LT-IIc (Figure 5C), suggesting that modulation of autophagy is involved in the selective LT-IIc-induced death of TNBC cells. To determine if the increase in the level of LC3B-II was due to induction and/or inhibition of autophagy, levels of p62/SQSTM1, a protein that undergoes autophagy dependent degradation [17], was assessed in MDA-MB-231 cells in response to LT-IIc. Figure 5D shows an increased level of p62/SQSTM1 protein following treatment of MDA-MB-231 cells, suggesting that LT-IIc is affecting autophagic flux by inhibiting autophagic progression. 

### 2.5. The Autophagy Inhibitor Bafilomycin A1 Blocks LT-IIc-Mediated Autolysosome Accumulation

To further investigate the capacity of LT-IIc to alter autophagic flux, MDA-MB-231 cells were treated with LT-IIc in the presence of bafilomycin A1, a drug that disrupts autophagic flux through inhibition of lysosome fusion with autophagosomes and decreasing lysosomal hydrolase activity [25,26]. Treatment of MDA-MB-231 cells with bafilomycin A1 or LT-IIc alone, each increased accumulation of LC3B-II protein as expected (Figure 6A,B). LC3B-II protein levels were not further enhanced by co-treatment of cells with LT-IIc and bafilomycin A1 (48 h). Interestingly, co-treatment blocked vacuole accumulation (Figure 6C), supporting the hypothesis that the enlarged vacuoles are autolysosomes formed by the fusion of autophagosomes with lysosomes [27]. Co-treatment of the cells with bafilomycin A1 enhanced the cytotoxic activity of LT-IIc (Figure 6D), suggesting that lysosome fusion and/or acidification are either already maximally blocked by LT-IIc or are not essential for toxin-mediated cell death. Similar effects were observed in cells by a 20 min pretreatment with bafilomycin A1 followed by LT-IIc for 4 h (data not shown). Collectively, these data support the hypothesis that LT-IIc inhibits autophagic flux through inhibition of autolysosomal trafficking and cargo degradation. Similar results were observed in BT-549 cells (Supplementary Figure S2).

### 2.6. LT-IIc Induces Autophagy through Inhibition of the mTOR Pathway

A recent study by Li et al. demonstrated that suppression of lysosomal acid hydrolase activity could paradoxically induce autophagy though inhibition of the autophagy-suppressive mTOR signaling pathway [28]. These findings, along with our observation that LT-IIc enhances levels of autophagy-associated proteins, prompted us to examine the phosphorylation status of p70S6K, a serine/threonine kinase that is downstream of the mTOR signaling pathway [29]. 

**Figure 7:**
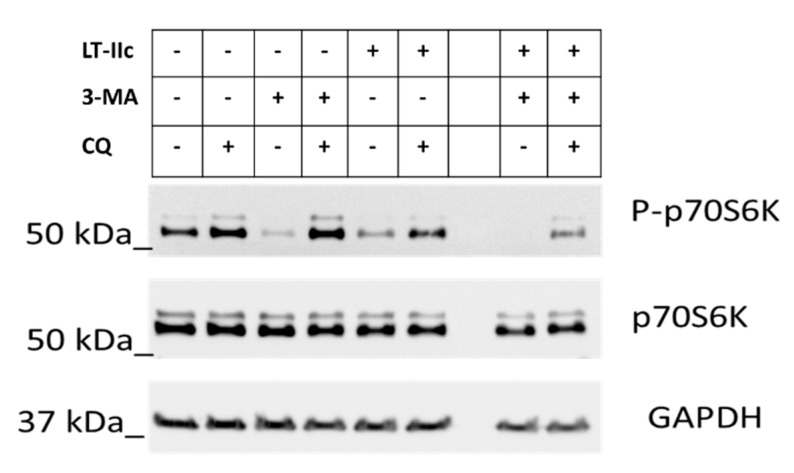
Effect of LT-IIc on p70S6K phosphorylation. MDA-MB-231 cells were treated with LT-IIc (5 µg/mL) ± 25 µM chloroquine (CQ) and/or 3-methyladinine (3-MA) (10 mM) as indicated. After 24 h treatment, cell lysates were harvested and analyzed by immunoblotting. Representative blot of three independent experiments.

In the absence of treatment, partial phosphorylation of p70S6K was observed, indicating partial mTOR activity and suppression of autophagy (Figure 7, Lane 1). Chloroquine (CQ), an inhibitor of autophagosome/lysosome fusion and acidification [30] enhances the phosphorylation of P70S6K (mTOR active/autophagy inactive) (Figure 7, Lane 2). As expected, treatment with 3-methyl adenine (3-MA), an activator of autophagy under nutrient-rich conditions via the PI3K pathway [31], dramatically decreases phosphorylation of p70S6K (Figure 7, Lane 3). Co-treatment with CQ and 3-MA completely restored mTOR signaling (Figure 7, Lane 4), suggesting that induction of autophagy by 3-MA synergizes with the blockage of autolysosomal fusion to reactivate mTOR. Treatment with LT-IIc resulted in effects similar to those of 3-MA alone, exhibiting a robust decrease in phosphorylated p70S6K, suggesting inhibition of mTOR and activation of autophagy (Figure 7, Lane 5). Combined treatment with 3-MA and LT-IIc further eliminated detectable phosphorylated p70-S6K, suggesting complete mTOR inhibition and induction of autophagy. Chloroquine blocked the effects of 3-MA (Figure 7, Lane 4), but only partially blocked LT-IIc-mediated inhibition of p70S6K phosphorylation (Figure 7, Lane 6). LT-IIc was able to partially overcome the activation of mTOR induced by combined treatment with 3-MA and CQ (Figure 7, Lane 4), evidenced by maintenance of partial dephosphorylation of p70S6K (Figure 7, Lane 9). 

### 2.7. LT-IIc Induces Apoptotosis in MDA-MB-231 Cells 

**Figure 8:**
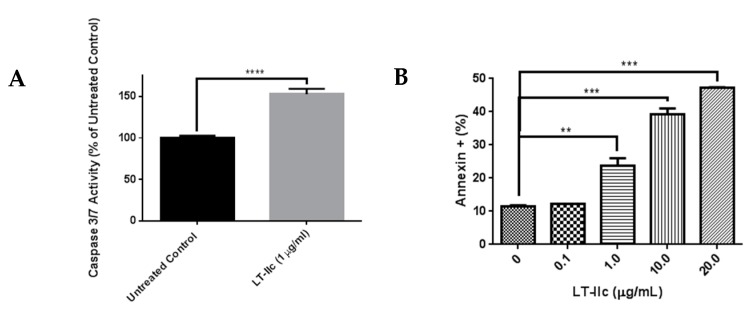
LT-IIc induces apoptotic cell death in TNBC cells. (**A**) MDA-MB-231 cells were treated with LT-IIc (1 µg/mL) prior to assessment of caspase 3/7 activity using a fluorescent substrate. Representative of >3 replicates from 3 independent experiments. (**B**) MDA-MB-231 cells were treated with 0, 0.1, 1.0, 10, or 20 µg/mL of LT-IIc for 24 h. Annexin V-stained cells were interrogated by flow cytometry within 1 h of staining. Bars represent means ± SEM. Key: ** *p* < 0.01; *** *p* < 0.001; **** *p* < 0.0001.

Apoptosis is a common mechanism of cell death that is induced by a variety of signals [32]. To test the hypothesis that LT-IIc reduced viable cell number by inducing apoptotic cell death, caspase 3/7 activity [33] was analyzed in cells treated with LT-IIc. Treatment of MDA-MB-231 cells with LT-IIc (1 µg/mL) elicited a ~50% increase in caspase 3/7 activity (Figure 8A). To determine if induction of apoptosis was toxin dose-dependent, annexin V staining, a hallmark of apoptosis induction, was examined. An increase in annexin V staining, in a dose dependent manner, was observed in TNBC cells treated with LT-IIc (Figure 8B). Similar results were observed in BT-549 cells (Supplementary Figure S3).

### 2.8. LT-IIc-Induced Cell Death is Mediated by a Combined Induction of Apoptosis and Necroptosis

**Figure 9:**
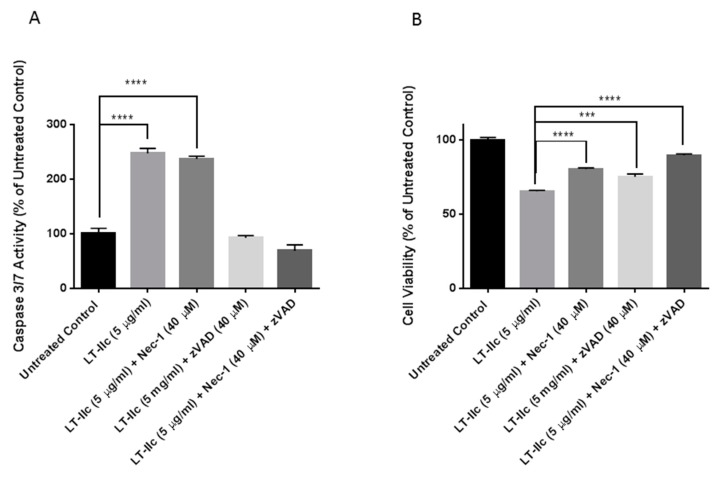
LT-IIc induces cell death through combined induction of apoptosis and necroptosis. MDA-MB-231 cells were treated with LT-IIc (5 µg/mL) in the presence or absence of Z-VAD-FMK (40 µM) and/or necrostatin-1 (40 µM) for 48 h. (**A**) Following treatment, caspase 3/7 activity was assessed using Apo-ONE^®^ Homogeneous Caspase-3/7 assay. (**B**) Cell viability was determined using MTT assay. Bars represent means ± SEM. Key: *** *p* < 0.001; **** *p* < 0.0001.

To investigate if the robust induction of apoptosis by LT-IIc is solely responsible for cell death, MDA-MB-231 were co-treated with LT-IIc in the presence or absence of Z-VAD-FMK, a pan-caspase inhibitor [34], and/or necrostatin-1, a necroptosis inhibitor [19] (Figure 9). Figure 9A shows that LT-IIc alone increased caspase 3/7 activity. As expected, caspase 3/7 activation (apoptosis) was not blocked by necrostatin-1, but was blocked by the caspase inhibitor Z-VAD-FMK. Overall cell viability, measured by mitochondrial activity using MTT, was assessed in Figure 9B. Treatment of toxin-exposed cells with either Z-VAD-FMK or necrostatin-1 rescued cytotoxicity. Co-treatment with both z-VAD-FMK and necrostatin-1 enhanced cytotoxic rescue. These results strongly indicated that the cytotoxic effect of LT-IIc is mediated through combined induction of both apoptosis and necroptosis.

### 2.9. ATG5 Knockdown Does Not Block LT-IIc-Mediated Cytotoxicity or Levels of LC3B-II

**Figure 10:**
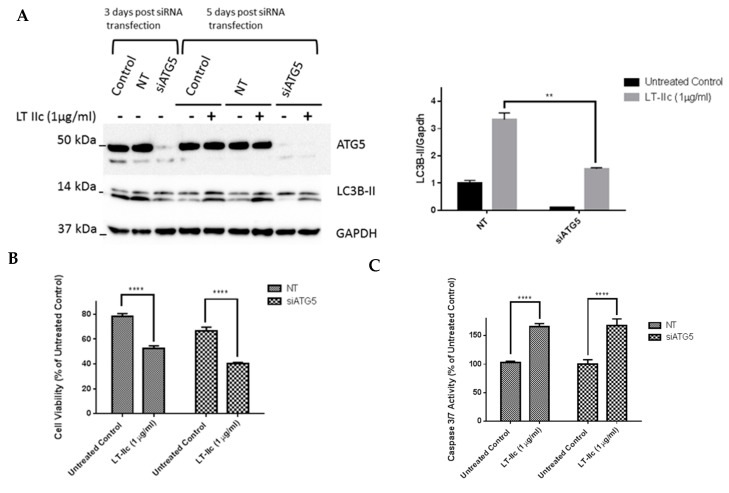
ATG5 gene knockdown using siRNA does not reverse LT-IIc effect on cytotoxicity or LC3B-II levels in MDA-MB-231 cells. (**A**) MDA-MB-231 cells were cultured in six-well plates prior to transfection with either ATG5 specific siRNA or a negative control NT siRNA. Three days post-transfection, ATG5 knockdown was confirmed using Western blotting. At day 5, another set of cells was treated with LT-IIc for 24 h to assess the requirement for ATG5 on LT-IIc-mediated increase in LC3B expression. The blots were quantified using ImageJ software. (**B**) Following the confirmation of ATG5 knockdown, the cells were cultured in 96-well plates prior to treatment with 1 µg/mL LT-IIc for 48 h. Cell viability was assessed using MTT assay, as described previously. (**C**) Following the confirmation of ATG5 knockdown, the cells were cultured in 96-well plates prior to treatment with 1 µg/mL LT-IIc for 24 h. Caspase 3/7 activity was determined using the fluorescent apo-One homogenous caspase 3/7 activity assay, as described above. All experiments are representative of at least two independent experiments. Error bars represent SEM. Key: ** *p* < 0.01, **** *p* < 0.0001.

To further examine the role of autophagy in LT-IIc-mediated cytotoxicity, siRNA-mediated knockdown of ATG5, a protein essential for autophagophore formation [35], was employed. ATG5 knock-down was confirmed by Western blotting three days post transfection (Figure 10A). A scrambled non-targeting (NT) siRNA was used as a negative control. Decreased levels, but not complete loss, of the lipidated form of LC3B (LC3B-II) was observed in cells in which ATG5 was silenced. Both LC3B-I and LC3B-II bands can be seen in Figure 10, with the antibody detecting, to a lesser degree, the higher molecular weight LC3B-I in addition to the stronger signal from LC3B-II.

Three days post-transfection, MDA-MB-231 cells with confirmed ATG5 knockdown were treated with LT-IIc. Unexpectedly, knockdown of ATG5 did not block the ability of LT-IIc to increase the levels of LC3B-II (lower band in Figure 10A,B). Since the lipidation of LC3B is proposed to be ATG5-dependent, these results suggest that LT-IIc either acts through a noncanonical mechanism to lipidate LC3B, or that the levels of LC3B-II in LT-IIC treated cells remain intact due to downstream effects, such as blockage of lysosomal degradation of the lipidated form. Overall cell viability assessed using MTT assay at 48 h showed that ATG5 knockdown did not block the cytotoxic effects of LT-IIc (Figure 10C). Analysis of caspase 3/7 activity revealed that LT-IIc induced apoptotic cell death independently of ATG5 protein expression (Figure 10D). These results further confirm that LT-IIc mediates its cytotoxic effects either through a pathway downstream of ATG5 or independent of the canonical ATG5 pathway. 

## 3. Discussion

The prognosis of TNBC patients remains poor due to the lack of effective targeted therapies and the recurrent and aggressive nature of the disease. There is a burgeoning interest in utilizing the properties of bacteria [36,37,38], bacterial products [39], and oncolytic viruses [40,41,42] to develop novel approaches to cancer therapy. Microbial products were at the forefront of cancer therapy as early as the 1890s, with the advent of the immune stimulating Coley’s Toxins. In addition to immune stimulation, direct mechanisms to target cancer are under investigation [3,36,43]. A number of microorganisms and their products have been shown to perturb the autophagic pathway [44,45]. Interestingly, one of the most effective microbial treatments used, Bacillus Calmette-Guerin (BCG) therapy for bladder cancer, has been shown to require an intact autophagic pathway for its efficacy [46]. 

The type I and II bacterial heat-labile enterotoxins are composed of a single catalytic A subunit that is non-covalently bound to a B pentamer. While the B pentamer binds to cell surface gangliosides, the A subunit ADP ribosylates the Gs subunit, which subsequently activates the host cell’s adenylate cyclase to dramatically elevate the levels of intracellular cAMP [47]. The results reported herein demonstrated that the cytotoxic effects of LT-IIc for human TNBC cells are not mediated through the A subunit-dependent activation of adenylate cyclase. This model was confirmed by demonstrating that other HLTs with the same enzymatic properties (e.g., CT, LT-IIa, and LT-IIb) did not induce a cytotoxic response. Furthermore, treatment of cells with forskolin, a direct activator of adenylate cyclase, did not mimic the cytotoxic effects of LT-IIc. These data strongly support the model that the cytotoxic effects of LT-IIc are mediated solely by binding of the enterotoxin to the TNBC cell surface. We surmise that, in comparison to other types of breast cells, TNBC cells uniquely or predominantly express one or more gangliosides that are bound by LT-IIc, a situation that would explain the specificity of LT-IIc for TNBC cells. Experiments are ongoing to determine if treatment of TNBC cells with LT-IIc-B5, a recombinant B pentamer that lacks the A polypeptide, has the same cytotoxic properties as LT-IIc holotoxin. If so, it will be critical to identify the ganglioside(s) bound by LT-IIc-B5 that initiate the cytotoxic responses in TNBC cells.

Recently, autophagy has emerged as a potential target for the development of novel breast cancer therapies. Dysregulation of autophagy impinges on multiple critical pathways involved in the development and progression of breast cancer, including hypoxia, genomic instability, oxidative stress, ER stress, bioenergetics (mitochondrial metabolism and the glycolytic shift), exosome formation, angiogenesis, and evolution of the tumor and its associated stroma [8,48,49,50,51,52,53,54,55,56,57]. Activated autophagic flux enables cells to resist detachment-associated cell death (anoikis resistance), which is a critical early step for the development of distal metastases [58]. The development of strategies to target autophagy in cancer has been confounded by the observation that autophagy can serve pro-survival or pro-death functions, depending on cellular context [59]. Accumulating evidence suggest that autophagy is altered by many cancer therapies including antimetabolites, paclitaxel, etoposide, vinblastine, NF-κB inhibitors, tyrosine kinase inhibitors, COX-2 inhibitors, estrogen signaling antagonists, angiogenesis inhibitors, DNA damaging agents and alkylating agents [37,38]. It should be recognized, however, that cellular heterogeneity within tumors and cell culture models could be a contributing factor to the controversial roles of autophagy in cancer [60,61]. 

The results reported herein demonstrated that LT-IIc stimulated autophagy by utilizing several distinctive cellular mechanisms including an inhibition of autophagic progression that caused an accumulation of autolysosomes. This accumulation of these LAMP-2-stained vesicles occurred within 3 h of initiation of treatment with LT-IIc. Analysis of the levels of LC3B-II and p62, normally degraded during autophagic progression after fusion of the autophagosome with the lysosome, indicated that LT-IIc likely disrupts autophagic progression by stabilizing the autolysosomes. This model is further supported by the observation that treatment of TNBC cells with bafilomycin A1, a drug which inhibits autophagosome/lysosomal fusion [27], blocks the capacity of LT-IIc to induce autolysosomes. Accumulation of the autolysosomes was accompanied by an inhibition in the mTOR signaling pathway, which can further induce autophagy. This model is in agreement with a recent study by Li et al. [28] in which suppression of lysosomal function induces autophagy via down-regulation of MTOR complex 1. The dominant effects of LT-IIc to suppress mTOR, even in the presence of the combined effects of 3-MA and CQ, suggest that LT-IIc can interrupt the feedback loop of these combined treatments. These results suggest that LT-IIc may initially inhibit autophagic flux through suppression of autolysosomal progression; in the additional presence of chloroquine, critical accumulation of autophagosomes in the absence of lysosomal fusion/acidification may feedback and partially re-activate the mTOR pathway (increased phosphorylation of p70S6K) [28]. The ability of CQ treatment to block the inhibitory effect of 3-MA on p70S6K phosphorylation is supported by a study by Wu et al. that demonstrated that 3-MA could act as autophagy inducer under nutrient rich conditions when the treatment is performed for long period of time (>9 h) [24]. In our studies, cells were exposed to 3-MA for 24 h under nutrient rich conditions. The combined stimulation of autophagy in response to 3-MA, and inhibition of lysosomal progression by CQ may have allowed critical overaccumulation of autophagosomes to provide a feedback loop to reactivate mTOR signaling and inhibit autophagy. 

Genetic experiments that knocked down expression of ATG5 further confirms that LT-IIc acts downstream and/or independently of ATG5 to inhibit autophagic progression through lysosomal degradation, as evidenced by the maintenance of LC3B-II levels. The retention of lipidated LC3B-II protein, in the absence of the ATG5 protein required for its generation by conjugation of LC3B with phosphatidylethanolamine, further supports the model that LT-IIc blocks lysosomal fusion and/or degradation of autophagosomal cargo (including LC3B-II). Interestingly, in the absence of autophagophore formation (which requires ATG5), cytotoxicity is maintained. These results suggest that LT-IIc can potentially induce cytotoxicity in autophagy dependent cell types through a dual mechanism, by enhancing the induction of autophagy, and/or by inhibiting lysosomal progression, resulting in “lysosome overload”. 

Analysis of caspase 3/7 activity in TNBC cells demonstrated that LT-IIc induces apoptosis. Co-treatment of MDA-MB-231 cells with LT-IIc in the presence of Z-VAD-FMK, a pan-caspase inhibitor, partially rescued cell death. Interestingly, treatment with LT-IIc in the presence of necrostatin-1, a known inhibitor of necroptosis, further enhanced the rescue of cell viability. Co-treatment of cells in the presence of both z-VAD-FMK and necrostatin-1 further increased cytotoxic rescue, thus demonstrating that LT-IIc ultimately causes cell death through a combination of apoptosis and necroptosis. The involvement of necroptosis in LT-IIc induced cell death could be due to accumulation of large number of autophagosomes and/or autolysosomes that serves as a scaffold for the components of the necroptosis machinery [62].

Several factors could contribute to the differential sensitivity of breast cancer subtypes to autophagy inhibition by LT-IIc. The association of the autophagy-dependent phenotype with TNBC cell lines may be related to the observation that productive autophagy is essential for survival of TNBC cells [8]. Additionally, breast cancer stem cells, which mediate resistance and relapse, are dependent on activated autophagy for survival [9]. The heterogeneity of breast cancer cell lines in terms of cancer stem cell abundance and cellular hierarchy in culture [63,64,65] may result in differential sensitivity of different breast cancer cell lines to the autophagy-altering effects of LT-IIc [66,67,68]. As noted above, the selective cytotoxic effects of LT-IIc could also result from binding to specific ganglioside receptors preferentially expressed on TNBC cells. While all four HLTs screened intoxicate cells via a similar ADP-ribosylating activity, each has a distinct ganglioside binding profile [13,69]. Ganglioside expression levels and patterns differ between different types of breast cancer cells. For instance, the expression level of total gangliosides is four fold higher in MDA-MB-231 cells in comparison to the total ganglioside composition of MCF7 cells [70]. Similarly, the specific level of GM3, the precursor to GM1 and most gangliosides, was found to be 18-fold higher in MDA-MB-231 cells [70]. In human breast cancer tissue, total ganglioside levels are elevated in invasive breast tumors in comparison to normal breast tissue [71]. Experiments are ongoing to identify the uniquely or predominantly expressed gangliosides on the surface of MDA-MB-231 and BT549 cells to establish their role in LT-IIc specificity. 

Collectively, the studies described herein demonstrated that LT-IIc acts as a bifunctional agent in autophagy-dependent TNBC by a lethal induction of autophagy and simultaneous inhibition of autophagic progression. Additionally, LT-IIc is exploitable as an experimental tool for identifying novel “druggable” targets and for revealing the gangliosides that could be employed as to biomarkers for TNBC diagnosis and as signal transducers for cell-specific cytotoxicity in cancer treatment. These effects likely extend beyond breast cancer, since preliminary experiments have shown similar cytotoxic effects of LT-IIc on melanoma cells. 

## 4. Materials and Methods 

### 4.1. Cell Lines and Reagents 

The breast cancer cell lines MDA-MB-231, MCF7, T47D, SKBR3, BT-549 and the non-transformed mammary epithelial MCF10A cells were obtained from the ATCC (Manassas, VA, USA) (CRM-HTB-26, HTB-22, HTB133, HTB30, HTB122 and CRL-10317, respectively). MDA-MB-231 cells were cultured in RPMI 1640 or F12/DMEM culture medium supplemented with 10% fetal bovine serum (FBS) and 1% penicillin/streptomycin. MCF7 cells were cultured in DMEM or F12/DMEM culture medium supplemented with 10% FBS and 1% penicillin/streptomycin. MCF10A cells were cultured in modified Eagle medium or F12/DMEM supplemented with 5% horse serum (Cat.# 35-030-CV), 20 ng/mL EGF (Cat.# E9644), 0.5 mg/mL hydrocortisone (Cat.# H0888), and 10 µg/mL insulin (Cat.# I1882). All media additives were purchased from Sigma-Aldrich (St. Louis, MO, USA), except basal media and 1% penicillin/streptomycin (Thermo-Fisher, Grand Island, NY, USA). Cells were grown at 37 °C in the presence of 5% CO_2_. Bafilomycin A1, chloroquine, and 3-methyladenine were obtained from Sigma-Aldrich. Antibodies to GAPDH (Cat.# SC25778) and p62 (Cat.# SC28359) were purchased from Santa Cruz Biotechnology (Dallas, TX, USA). ATG5 antibody (Cat.# 12994) and LC3B antibody (Cat.# 3868S) were purchased from Cell Signaling Technologies (Danvers, MA, USA). The LC3B (D11) XP^®^ Rabbit mAb (Cell Signalling Technologies, Cat.# 3868) can detect total LC3B protein, i.e., precursor LC3B-I and lipidated LC3B-II. Its reactivity with lipidated LC3B-II is substantially greater than with unmodified LC3B-I (https://www.cellsignal.com/products/primary-antibodies/lc3b-d11-xp-rabbit-mab/3868). 

MTT cell viability assay (Cat.# G4100) and caspase 3/7 activity assay (Cat.# G7790) were purchased from Promega (Madison, WI, USA). Recombinant LT-IIc was purified from *E. coli* using nickel affinity and gel chromatography, as described previously [5]. ATG5 siRNA (SMARTpool ON-TARGETplus, Cat.# L-004374-00-0005) and non-targeting siRNA (ON-TARGETplus Non-targeting Pool, #Cat.# D-001810-10-05) were purchased from Dharmacon (Lafayette, CO, USA). Z-VAD-FMK (Cat.# ALX-260-020) was purchased from Enzo (Farmingdale, NY, USA) and necrostatin-1 (Cat.# 11658) was purchased from Cayman chemicals (Ann Arbor, MI, USA).

### 4.2. Cell Viability and Caspase 3/7 Activity Assays 

MTT cell viability assay was conducted using CellTiter 96^®^ Non-Radioactive Cell Proliferation Assay kit (Promega, Cat.# G4100). Briefly, the cells were cultured overnight in 96-well plates at density of 2 × 10^3^ cells/well and treated with the appropriate drug for 24 to 48 h before addition of the MTT reagent. Caspase 3/7 activity was measured using Apo-ONE^®^ Homogeneous Caspase-3/7 Assay kit (Promega, Cat.# G7792). Briefly, cells were cultured overnight in 96-well plates and treated with the appropriate drug on the following day for 24 h. Caspase 3/7 substrate was added and the plate was incubated at RT for up to 18 h before measurement of the fluorescent signal using a 96-well BioTek Synergy HT plate reader (Winooski, VT, USA). 

### 4.3. Treatment with ZVAD-FMK and Necrostatin-1

MDA-MB-231 cells were plated at 5 × 10^4^ cells in basal media in 96-well plates. After overnight incubation to allow adhesion, while cells were still in logarithmic phase, media was supplemented with ±LT-IIc 5 µg/mL, ±(40 µM) Z-VAD-FMK, ±(40 µM) necrostatin-1. After 48 h of treatment, cells were assayed for caspase 3/7 activity and MTT uptake. 

### 4.4. Annexin V-FITC Apoptosis Assay

MDA-MB-231 cells were plated in 6-well plates at 1 × 10^5^ cells/well in 2 mL of media. After overnight adhesion, while still in logarithmic phase, cells were treated for 24 h with 0, 0.1, 1.0, 10, or 20 µg/mL of LT-IIc. Cells were harvested into 1 mL of 0.25% trypsin/EDTA and incubated for 3 min at 37 °C. Cells were washed 2× in 3 mL of ice-cold PBS, centrifuged at 300× *g* for 5 min at 4 °C, and resuspended in 100 µL of 1× staining buffer containing 5 µL of Annexin V FITC. After 15 min at RT, 400 µL of 1× staining buffer was added to each sample. Within 1 h of staining, cells were interrogated using an LSR Fortessa (BD Biosciences, San Jose, CA, USA). 

### 4.5. Western Blotting

Cells in log phase of growth were cultured overnight in 6-well plates prior to drug treatment. To harvest protein, cells were washed 2× with ice-cold PBS and lysed using CelLytic M lysis buffer (Sigma-Aldrich, Cat.# C2978). Protein concentrations were measured using a Bradford protein assay (Bio-Rad, Hercules, CA, USA). A total of 30 µg of protein/sample was loaded in each lane, resolved using SDS-PAGE, and transferred to PVDF membrane (Bio-Rad Cat.# 1620177), after which the membranes were immunoblotted using target-specific antibodies. Chemiluminescence imaging was performed using a FluorChem M imager (Cell Biosciences, Inc., San Jose, CA, USA). Densitometry was performed using ImageJ software (NIH, Bethesda, MD, USA).

### 4.6. Oil Red O Staining

MDA-MB-231 and BT549 cells were plated in 6-well plates at 5 × 10^4^ cells/mL, and allowed to adhere overnight before replacing media with fresh media ±1 µg/mL LT-IIc. Cells were treated for 24 h followed by fixation with 4% phosphate buffered formalin, 2 mL per well, for 10 min. Wells were rinsed with distilled water, then 60% isopropanol for 2–5 min. Fresh Oil Red O (Sigma, Cat.# O-0625) was prepared immediately from a stock solution of 0.3% Oil Red O (*w*/*v*) in 99% isopropanol. Immediately before use, the 0.3% Oil Red O solution was further diluted at a 3:2 ratio with distilled water and filtered using #1 Whatman filter paper. Oil Red O was applied for 5 min, followed by 3× rinses with 2 mL/well of dd H_2_O. Cells were photographed in situ in the plate using an Olympus BH2 inverted microscope (Waltham, MA, USA) with a Moticam 2.0 digital camera, under bright field illumination to see Oil Red O, and phase contrast, to visualize cellular vacuoles. 

### 4.7. LAMP-2 Staining

MDA-MB-231 were plated at 5 × 10^4^ cells/mL on sterile glass coverslips in 6-well plates. The next day, while cells were still in logarithmic phase, cells were treated with 0 or 5 µg/mL LT-IIc holotoxin for 6 h. Cells were fixed in 3.7% phosphate buffered formalin and stored at 4 °C in sterile PBS until stained. Immunofluorescent staining was performed with 1 µg/mL DAPI (Sigma Cat.#D9542) and 1:100 mouse anti-LAMP-2 (Novus, Cat.# NBP2-22217 (Centennial, CO, USA), followed by goat anti-mouse Alexa 594. Images from control and treated cells were photographed using the same manual exposure times under a 40× objective using a Zeiss Axioimager microscope with Axiocam (San Diego, CA, USA). 

### 4.8. Cell Imaging

For phase contrast images, cells were seeded at 5 × 10^4^ cells/mL in 6-well plates and allowed to attach overnight. Following 24 or 48 h-treatment with LT-IIc in the presence or absence of various pharmacological agents. The cells were imaged using an EVOS FL Cell Imaging System imager purchased from ThermoFisher Scientific (Waltham, MA, USA). Autophagic vacuole size was measured in MDA-MB-231 and BT549 cells treated for at least 24 h with 1 µg/mL LT-IIC, using 40× objective images from an Olympus BH2 microscope (Olympus Life Sciences, Waltham, MA, USA). The largest diameter vacuole (one per cell) in multiple cells was measured along the longest diameter, and this value was averaged with the measurement at 90 degrees. The millimeter measurements were converted to microns using a stage micrometer image. Measurements were compared from at least three independent experiments. 

### 4.9. Cell Transfection with siRNA

MDA-MB-231 cells were cultured overnight in six-well plates prior to transfection using Dharmafect 4 transfection agent (Dharmacon, Cat#: T-2004-01), following the manufacturer’s protocol. After 72 h, cells were immunoblotted to confirm ATG5 knockdown. Cells plated in parallel were used or MTT and caspase 3/7 activity assays to assess the efficacy of LT-IIc in ATG5 knockdown cells in comparison to cells transfected with a scrambled control siRNA. 

### 4.10. Statistical Analysis

Data were analyzed using Graphpad Prism software (GraphPad Prism Inc., La Jolla, CA, USA). Statistical significance of the data was determined using Students’ *t*-test, one-way, or two-way ANOVA analysis, as appropriate.

## Supplementary Materials

The supplementary materials are available online at http://www.mdpi.com/1422-0067/20/1/85/s1.
