# Supplementary material for: LT-IIc, A Bacterial Type II Heat-Labile Enterotoxin, Induces Specific Lethality in Triple Negative Breast Cancer Cells by Modulation of Autophagy and Induction of Apoptosis and Necroptosis
Source: Int J Mol Sci. 2018 Dec 26;20(1):85. doi: 10.3390/ijms20010085 (PMC6337683; doi:10.3390/ijms20010085)
Supplement: Supplementary file 1 [file ijms-20-00085-s001.pdf]

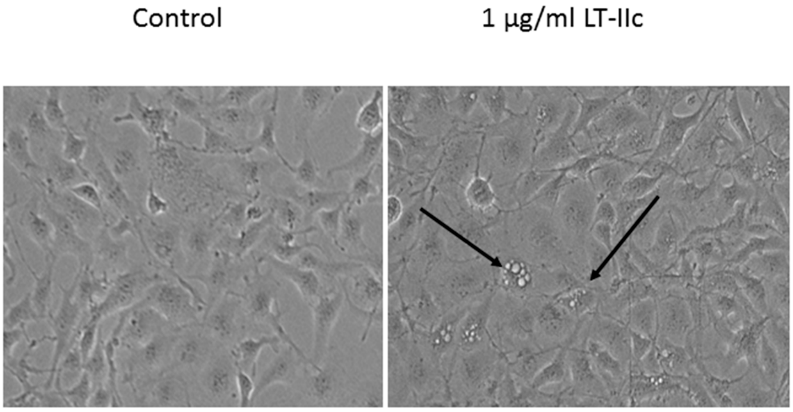

Figure S1. BT-549 cells were treated with 1 μg/ml LT-IIc for 24 hr. Cell images were obtained using 40X objective. Arrows indicate vacuolation in BT-549 cells.

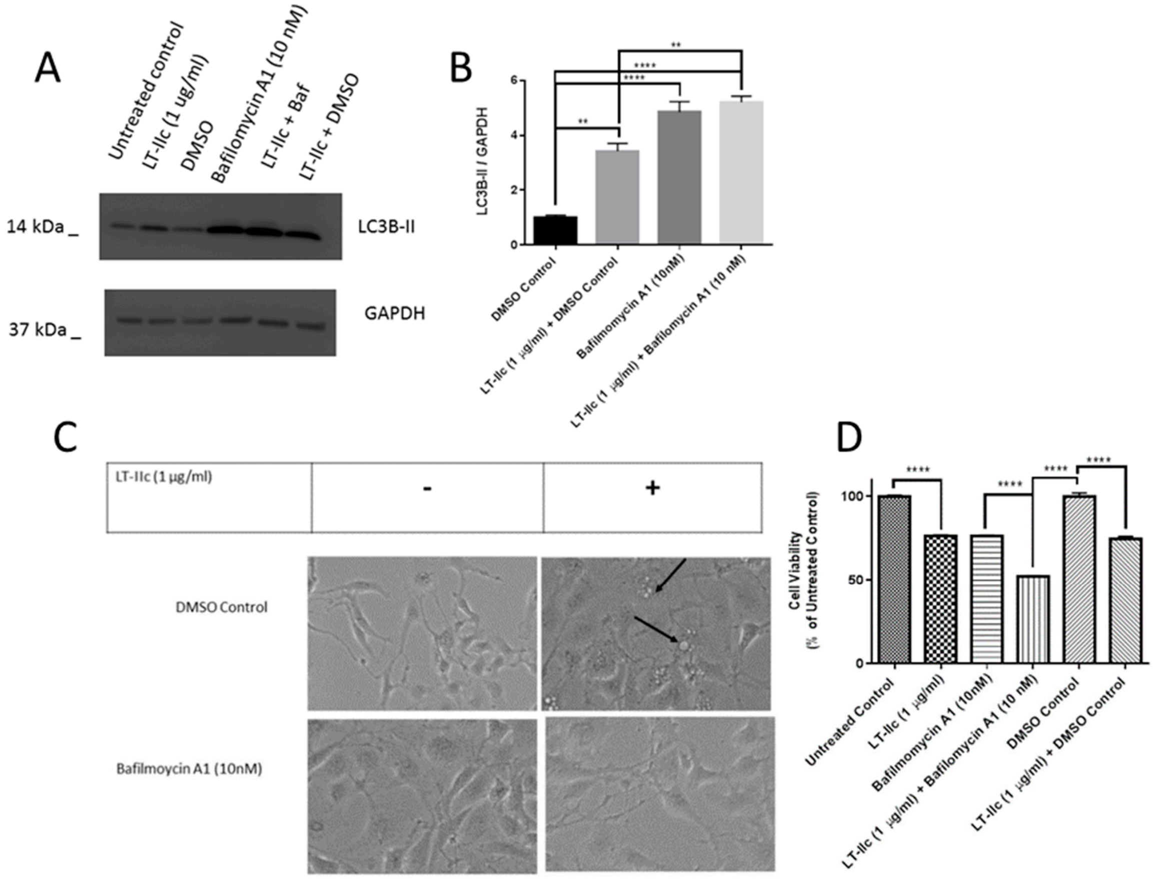

Figure S2. Bafilomycin A1 enhances cytotoxicity of LT-IIc efficacy in BT-549 cells. (**A**, **B**) BT-549 cells were treated with LT-IIc (1 μg/mL) +/- bafilomycin A1 (10 nM) for 48 h. Expression of LC3B-II was analyzed using Western blotting, and quantified using ImageJ. (**C**) BT-549 cells were treated with LT-IIc (1 μg/mL) in the presence or absence of bafilomycin A1 (10 nM) for 24 h and cell morphology was evaluated using microscopic analysis (40× objective). (**D**) BT-549 cells were treated with LT-IIc (1 μg/mL) +/- bafilomycin A1 (10 nM) for 48 h and cell viability was analyzed using MTT assay. Images represent at least three independent replicates from at least two independent experiments. Error bars represent standard error of the mean. **, p <0.001; ****, p <0.00001.

A B

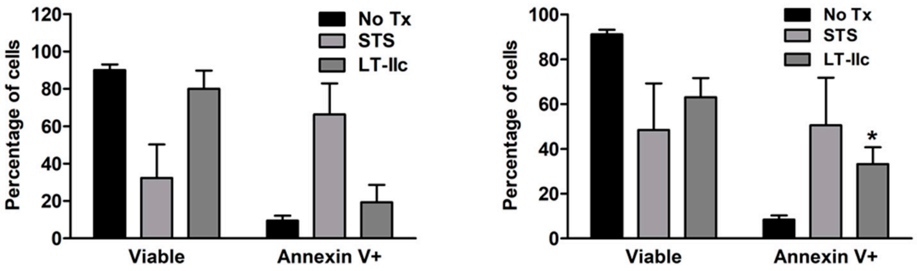

Figure S3. LT-IIc induces apoptosis in TNBC MDA-MB-231 (**A**) and BT-549 cells (**B**). The cells were plated in six-well plates at 1 × 10^5 cells/well in 2 mL of media. After 48 h, cells were treated LT-IIc (1 μg/mL) or staurosporine (STS) (1 μM) for 24 h, and stained for Annexin V/PI binding. Data flow cytometric analysis of % viable (negative for Annexin V and PI), apoptotic (Annexin V positive), or dead/necrotic (PI positive or dual positive) cells. *, $p < 0.05$ difference from control.
